# Preparation of Calibration Standards of N1-H Paralytic Shellfish Toxin Analogues by Large-Scale Culture of Cyanobacterium *Anabaena circinalis* (TA04)

**DOI:** 10.3390/md9030466

**Published:** 2011-03-22

**Authors:** Ryuichi Watanabe, Toshiyuki Suzuki, Yasukatsu Oshima

**Affiliations:** 1 National Research Institute of Fisheries Science, 2-12-4 Fukuura, Kanazawa-ku, Yokohama 236-8648, Japan; E-Mail: tsuzuki@affrc.go.jp; 2 School of Marine Biosciences, Kitasato University, 160-4 Okirai-uto, Sanriku-cho, Ofunato 022-0101, Japan; E-Mail: oshima.y@kitasato-u.ac.jp

**Keywords:** cyanobacteria, *Anabaena circinalis*, paralytic shellfish toxin, saxitoxin, chemical derivatization, standard

## Abstract

Mouse bioassay is the official testing method to quantify paralytic shellfish toxins (PSTs) in bivalves. A number of alternative analytical methods have been reported. Some methods have been evaluated by a single laboratory validation. Among the different types of methods, chemical analyses are capable of identifying and quantifying the toxins, however a shortage of the necessary calibration standards hampers implementation of the chemical analyses in routine monitoring of PSTs in bivalves. In our present study, we studied preparation of major PST analogues as calibrants by large-scale cultivation of toxic freshwater cyanobacteria *Anabaena circinalis* TA04. The cells were steadily grown in 10 L bottle for 28 days. The primary N1-H toxins, C1/C2, were produced at a concentration of 1.3 ±0.1 μmol/L. The intracellular and extracellular toxins occupied 80% and 20%, respectively. Over 220 μmol of the toxins was obtained from approximately 200 L of the culture over six months, demonstrating that it is sufficient to prepare saxitoxin analogues. The toxins were chemically converted to six N1-H analogues. Preparation of the analogues was carried out at relatively high yields (50–90%). The results indicate that our preparation method is useful to produce N1-H toxins. In our present study, detailed conditions for preparation of one of the rare N1-H analogues, gonyautoxin-5, were investigated.

## 1. Introduction

**Figure 1.**
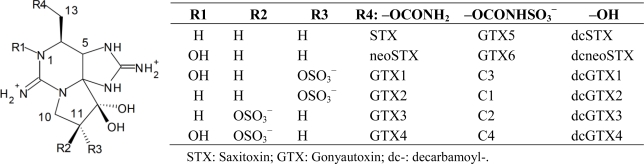
Structures of paralytic shellfish toxins.

Paralytic shellfish toxins (PSTs), one of the most potent groups of neurotoxins in marine biotoxins, are produced by toxic marine dinoflagellates and freshwater cyanobacteria. Around 30 analogues have been reported (Figure 1). Some of these analogues are metabolic products of algae-derived toxins in shellfish [1]. Approximately 20 analogues are found to show a wide range of toxicity [2]. Natural and cultivated shellfish are contaminated with PSTs by feeding on toxin-producing algae, and are a potential cause of paralytic shellfish poisoning (PSP) to humans. Although mouse bioassay (MBA), which is the official testing method to determine shellfish toxicity in many countries, is effective in protecting the consumers from PSP, alternative methods are required due to some drawbacks including accuracy, reproducibility and ethical concerns [3].

A number of alternative analytical methods have been developed to date. Some have been evaluated by a single laboratory validation [4,5]. Among different types of methods, high performance liquid chromatography fluorescent detection (HPLC-FD) is one of the reliable methods to identify and quantify individual toxins. It is reported that the toxicity obtained by HPLC-FD is usually consistent with the total toxicity of MBA by measuring the major toxins C1–4, gonyautoxin (GTX) 1–6, decarbamoylgonyautoxin (dcGTX) 2 and dcGTX3, neosaxitoxin (neoSTX), decarbamoylsaxitoxin (dcSTX) and saxitoxin (STX) [2]. However, although the calibration standards are commercially available from the National Research Council Canada (NRC), shortage of the calibration standards has hampered implementation of HPLC-FD in routine monitoring of PSTs in bivalves.

In preparation of high amount of the calibration standards, selection of natural source materials is important. Shellfish with a high level of toxicity [6,7] are one of the important natural sources but this source has hardly been obtainable in Japan over the last decade due to very low levels of toxicity (at most 10–50 MU/g). Therefore, some toxic dinoflagellates such as *Alexandrium excavatum* [8] and *A. tamarense* [9] are used as appropriate source materials in terms of stable toxin source materials. Some toxin-producing cyanobacteria such as *Anabaena circinalis* and *Aphanizomenon flos-aquae* would also be ideal source materials in toxin production and cell growth.

Because primary toxins produced by toxic algae are insufficient to account for the complicated PST analogues in shellfish, chemical conversion of the primary toxins to other analogues is used to produce a variety of toxins. Some chemical or enzymatic reactions were summarized for each substituent moiety; C11, C13 and N1 positions [10].

Among the known toxins, *N*-sulfocarbamoyl gonyautoxins, C1/C2, are useful to produce other N1-H analogues by chemically removing the substituent moieties. Although adequate conditions for conversion of C1/C2 to dcGTX2/dcGTX3, dcSTX, and GTX2/GTX3 were reported in previous papers [11,12], detailed investigation of chemical conversion of other analogues (e.g., GTX5) that are more dominant toxins in shellfish, has not been reported yet. In this paper, we focused on large-scale cultivation of the cyanobacteria *A. circinalis* to prepare a variety of N1-H analogues from C1/C2 by chemical conversion.

## 2. Results and Discussion

### 2.1. Large-Scale Culture of Cyanobacteria *A. Circinalis* (TA04)

**Figure 2.**
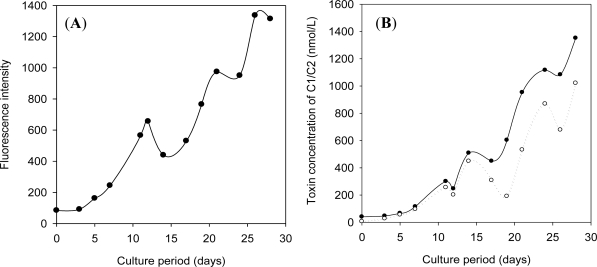
Growth curve (**A**) and toxin production (**B**) in a large-scale culture of *A. circinalis*. (**A**) Fluorescent intensity shows the average level of chlorophyll *a* in cells (*n* = 3); (**B**) Only C1/C2 produced by the cells were analyzed by high performance liquid chromatography fluorescent detection (HPLC-FD). Solid line shows total toxin concentration of C1/C2 in culture. Dashed line shows intracellular toxin concentration of C1/C2. The difference corresponds to the extracellular toxin concentration.

The cultured *A. circinalis* (strain TA04) has been maintained in modified C medium since 1996 [13]. The culture showed dark-green color in mid-stationary phase, and turned to chrome yellow color in late-stationary phase. The biomass and the toxin concentration also increased in the exponential growth phase, followed by the short induction growth phase (∼7 days, Figure 2). As the toxin concentration declined when the culture reached the late-stationary phase, the cells were harvested at 28 days. The cultured cyanobacteria produced C1/C2 (70–90%) as the dominant toxins. Although GTX2/GTX3 and dcGTX2/dcGTX3 (5–15% each) were produced as the minor toxins, N1-hydroxylated toxins such as GTX1/GTX4 were not found in the harvested culture. The toxin components determined in our present study were different from those obtained in our previous study [13].

This difference is probably caused by physiological natural changes due to the long-term successive culture over 15 years and the change of culture medium. On the other hand, the α/β-epimer ratio of C-toxins was 1.1, which was close to previous data [14]. The toxin production of C1/C2 in large-scale culture was 1.3 ± 0.1 μmol/L culture (7% RSD, *n* = 3) at 28 days. This value was somewhat lower than that found in our previous study (1.8–2.0 μmol/L culture). Approximately 80% of the total toxins were found inside the cells. Our result was similar to that obtained in another strain, MB06 [14], which was collected in the same location as our material strain, TA04, in the ratio of epimers and intra-/extracellular toxin in 1.2 L small scale culture, whereas the cell growth of strain TA04 was slower than strain MB06.

The cyanobacteria were cultured under several modified conditions of Fitzgerald medium toward efficient toxin production. The biomass and toxin production for 28 days were investigated in each condition (Table 1). As a result, the concentration of nitrate and phosphate hardly affected the toxin production and biomass of the cyanobacteria. Two different inoculation volumes were determined to shorten the culture period, but the cell growth and the toxin production were the same as those of the control. In PST biosynthesis of cyanobacteria, the precursors such as arginine and *S*-adenosylmethionine were identified by the cultivation with stable isotopes [12] and a hypothetical pathway was proposed. The *in vitro* experiments for PST biosynthesis suggested that pyridoxal phosphate (PLP)-dependent enzymes were strongly involved in PST biosynthesis [15]. Therefore, the toxin production in the cell was investigated *in vivo* by adding a common cofactor, PLP, to the medium. Our study showed PLP hardly affected both cell growth and toxin production in our culture. PLP could be insufficiently incorporated into cells to be available to enzymes.

Moreover, the addition of organic phosphates, β-glycerophosphate sodium salt of 10 and 100 μM each, was also investigated and no significant change was observed. Finally, Good’s buffer, bicine, was also used to control the pH of the culture medium during culture. The toxin production was slightly improved compared to the control, but the cell harvest was hampered and the intracellular toxins leaked outside in harvest because of the increased viscosity of the culture.

Figure 2 indicates that approximately 140 μmol of C1/C2 could be produced from 100 L culture. It was demonstrated that over 220 μmol of C1/C2 was obtained from 200 L of the culture which was produced over 6 months cultivation. This amount of toxins would be sufficient to prepare the calibration standards of STX analogues.

### 2.2. Preparation of Paralytic Shellfish Toxin Analogues

**Figure 3.**
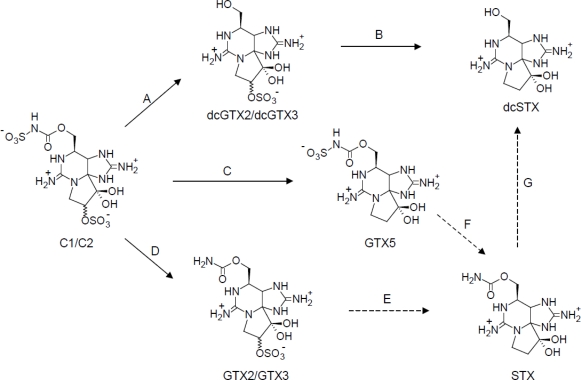
Preparation scheme of paralytic shellfish toxin analogues. (**A**) Phosphate buffer (pH 7.0), 70 °C, 60 min; (**B**) Phosphate buffer (pH 6.0), 2-mercaptoethanol, 60 °C, 60 min; (**C**) Acetate buffer (pH 5.2), 2-mercaptoethanol, 50 °C, 2 h; (**D**) 0. 13 M HCl aq., 100 °C, 15 min; (**E**) 2-mercaptoethanol, 100 °C, 15 min.; (**F**) 0.05 M HCl, 100 °C, 30 min [17]; (**G**) 7.5 M HCl, 100 °C, 3 h [18,19]. Solid arrows, this study; dashed arrows, previous studies.

C1/C2 are one of the most modified STX analogues among the known toxins. C1/C2 are also known as the lowest toxicity group among STX analogues [2]. Figure 3 shows the preparation scheme of PST analogues by chemical conversion from C1/C2. *N*-sulfocarbamoyl group on C1/C2 is hydrolyzed in neutral pH, and this reaction leads C1/C2 to dcGTX2/dcGTX3. Subsequently, the sulfate ester group of the toxins is reductively eliminated by 2-mercaptoethanol (2-ME) to prepare dcSTX [11]. Thus, our route to produce dcSTX is useful compared to other routes through STX [16] because production of STX is legally restricted in Japan and many countries.

**Figure 4.**
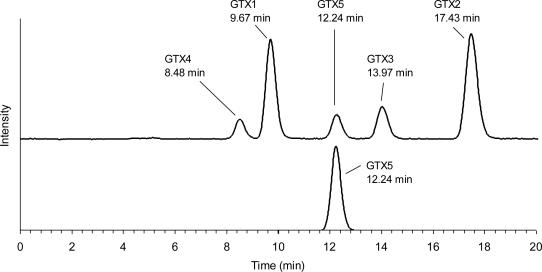
Chromatograms of the authentic GTX5 (upper) and the product (bottom). The authentic GTX5 was used as a mixture of GTX1–5.

GTX5 was directly converted from C1/C2 by reductive elimination of the sulfate. C1/C2 were reacted with 2-ME to give 48% of GTX5, 18% of the remaining C1/C2, and 34% of unknown reactants or the loss. Of the reaction temperatures tested (50–80 °C), a good yield (50%) of GTX5 was obtained at 50 °C. The yield reached 30% within 30 min and slowly increased with time, and then gradually declined over 3 h. The remaining substrate was decreased to 20–40% within 30 min and then slightly declined. HPLC analysis of the reactant mixture revealed that dcGTX2/dcGTX3 and dcSTX, as by-products in the reaction, were not produced, indicating that the reaction specifically proceeded without hydrolyzing the *N*-sulfocarbamoyl chain of C1/C2. Interestingly, HPLC analysis of the reaction mixture revealed that the relative proportion of C1 to C2 decreased after the reaction, implying that the α-epimer reacted selectively with a thiol compound compared to the β-epimer. Chromatographic behavior of the product was the same as that of the authentic GTX5 (Figure 4).

**Figure 5.**
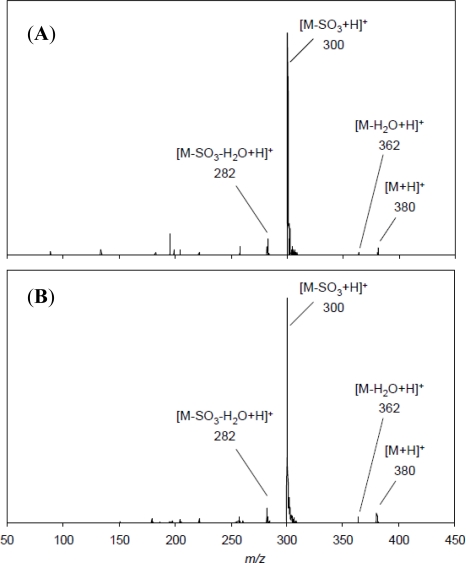
MS/MS spectra of the authentic GTX5 (**A**) and the product (**B**). Both toxins were analyzed at declustering potential of 11 V and collision energy of 21 V. The precursor ion was set to 380.

Figure 5 shows LC-MS/MS spectra of the authentic GTX5 and the product converted from C1/C2. The MS/MS spectrum of the authentic GTX5 gave ion peaks at *m/z* 282, and 300 corresponding to [M − H_2_O − SO_3_ + H]^+^ and [M − SO_3_ + H]^+^, respectively (Figure 5A). Basically the same fragment ions were obtained for the product (Figure 5B), demonstrating that the product was GTX5. GTX5 was prepared in NaHCO_3_ solution containing dithiothreitol (DTT) in a previous study [20]. They also attempted preparation of GTX5 by the reaction of C1/C2 with 2-ME in place of DTT, but GTX5 was not obtained. The fact that our method was successful to produce GTX5 with 2-ME was probably because of acidic condition in which the thioester intermediate obtained in neutral pH [21] was not formed. Conversion efficiency of GTX5 from C1/C2 prepared in our present method was better than that (36%) reported in the previous study [20].

The toxins with an *N*-sulfate group such as C3/C4, GTX5 and GTX6 are easily converted to the corresponding carbamate toxins by hydrolyzing in diluted mineral acids [12,19,22]. In our present study, this hydrolysis reaction was applied to prepare GTX2/GTX3 from C1/C2. The products showed the same chromatographic behavior with the authentic GTX2/GTX3 in HPLC-FD. The products, GTX2/GTX3, were obtained as an equilibrium mixture (ratio: 3.0 to 3.5), although C1/C2 used for the reaction were not an equilibrium mixture.

In LC-MS/MS analysis, epimers such as GTX2/GTX3 show different fragmentation patterns. GTX2 as α-epimer gives a predominant ion peak at *m/z* 316 corresponding to [M − SO_3_ + H]^+^, whereas GTX3 as β-epimer gives several major peaks at *m/z* 298, 316 and 378 corresponding to [M − H_2_O + H]^+^. The products were confirmed to show the same fragmentation pattern in each epimer.

In the process of preparation of PST analogues, it was a requirement to avoid generation of STX because production of STX is illegal in Japan and many countries. In our method, STX was not detected in any processes by HPLC-FD. Although STX is not prepared in this study as mentioned above, STX could be prepared from GTX2/GTX3 or GTX5 as described in the previous reports [12,17], if necessary. Thus, C1/C2 as starting materials could be converted to six analogues and STX, giving a relatively high yield in each reaction (Figure 3). The starting materials of C1/C2 can be obtained at a yield of 50% from the extract of the cell by purifying them with several chromatography steps such as activated charcoal, gel filtration, and ion exchange. The purified C1/C2 were converted to dcGTX2/dcGTX3 (step A), GTX5 (step C), and GTX2/GTX3 (step D), and subsequently a portion of the resulting dcGTX2/dcGTX3 were converted to dcSTX (step B). At least 10 μmol of the toxin calibrants could be prepared from 200 L of culture of the toxic cyanobacteria (Table 2).

## 3. Experimental Section

### 3.1. Large-Scale Culture of Cyanobacteria and the Harvest

The cyanobacterium *A. circinalis* used in this study is a non-axenic strain TA04. The field sample of *A. circinalis* was collected at Tullaroop reservoir, Victoria, Australia, and strain TA04 was one of single-trichome isolates prepared by Negri *et al.* [14]. Strain TA04 was cultured in Fitzgerald medium in 2 L culture, and then scaled-up to 10 L volume (Thermo Fisher Scientific, Rochester, NY, USA). The cyanobacteria were cultured on a 16 L/8 D cycle at 17 °C and 25 μmol/m^2^/s, passing air through a disc filter (0.20 μm pore size, Advantec, Japan). The bottle cap was equipped with three pores, where one was used as air-inlet with the disc filter, one was as air-outlet with the disc filter and one was as a sampling tube with a cock. The cyanobacteria were maintained by inoculating 1 L of the culture in exponential growth phase (two weeks later) to 9 L of the sterilized medium. The 10 L batch culture was carried out in quintuplicate, of which one was used for inoculation of seed population, and repeated three times. Four weeks later after inoculation of the seed population, the *A. circinalis* cells were harvested by a continuous centrifugation or filtration (No. 2, Advantec, Japan). The cell-free culture filtrate and the cell fractions were treated according to our previous procedure [11].

### 3.2. Cell Growth Measurement and Toxin Analysis

Cell growth was estimated from fluorescent intensity derived from chlorophyll *a* in the cell. The culture was taken in axenic through a sampling tube. Culture samples (200 μL) were collected, and added to wells of a 96-well microplate in triplicate. The cell-free culture filtrate (200 μL) as blank was prepared by filtering the culture with a syringe filter (Dismic-25cs, 0.45 μm, Advantec, Japan) or a glass fiber paper (GA-100, 25 mm, Advantec, Japan). It is subtracted from the culture fluorescence using a fluorescence plate reader (Ex: 460 nm; Em: 645 nm, FL600, BioTek).

Toxin content (C1/C2) of the cell and the cell-free filtrate was analyzed with HPLC-FD [2]. The culture of 10 to 30 mL at each growth phase was passed through a glass fiber paper (GA-100, 25 mm, Advantec, Japan) to collect cells. The paper was dissected and suspended into 1 mL of 0.5 M acetic acid. The suspension was centrifuged, following ultrasonication, to obtain the supernatant as analyte. On the other hand, the filtrate was adjusted to become acidic by adding 0.5 M acetic acid. The filtrate was evaporated to dryness and then dissolved in 500 μL of 0.5 M acetic acid. The toxin solution was ultrafiltered (Ultracell YM-10, 10000 MWCO, Millipore, Billerica, MA, USA) to obtain the resulting filtrate as analyte.

### 3.3. Serial Preparation of dcGTX2/dcGTX3 and dcSTX from C1/C2

The preparation of dcGTX2/dcGTX3 and dcSTX from C1/C2 was carried out according to the method of Watanabe *et al.* [11]. C1/C2 as starting materials were purified from the harvested cell fraction in the cyanobacteria *A. circinalis* (TA04) culture. Typically, dcGTX2/dcGTX3 were obtained by the hydrolysis of C1/C2. C1/C2 were heated in 100 mM potassium phosphate buffer at pH 7.0 for 60 min, stirring at 70 °C, and then cooled on ice. The resulting dcGTX2/dcGTX3 were isolated by gel filtration chromatography (Bio Gel P-2, 10 mm × 400 mm, Bio-Rad) and weak-cation exchange chromatography (Bio-Rex 70, 200–400 mesh, 10 mm × 400 mm, Bio-Rad) at overall yield of 50%. The purified dcGTX2/dcGTX3 was reacted under stirring conditions at 60 °C in 100 mM potassium phosphate buffer at pH 6.0 containing an equal volume of 2-ME for 60 min to quantitatively give the resulting dcSTX.

### 3.4. Preparation of GTX5 from C1/C2

C1/C2 (1.4 μmol) were evaporated to dryness and dissolved in 2 mL of 100 mM acetate buffer at pH 5.2 containing equal volume of 2-ME in a 4 mL glass vial. The toxin solution was reacted at 50 °C for 2 h. The reactant was evaporated and then diluted with acetonitrile to be a final concentration of 90% (v/v) acetonitrile solution and then the diluted solution was passed through a ZIC-HILIC cartridge (3 mL, 200 mg, Merck SeQuant, Umeå, Sweden) preconditioned with 6 mL of distilled water and 6 mL of acetonitrile in order. The cartridge was washed with 3 mL of acetonitrile and then eluted with 3 mL of distilled water as toxic fraction. The toxic fraction was purified by Bio-Rex 70 (200–400 mesh, 10 mm × 450 mm, Bio-Rad) to give GTX5 at an overall yield of 48%. The product was confirmed and identified by the comparison with the authentic standard that was previously prepared by Oshima and confirmed in structure and purity with NMR [2]. Chromatographic behavior of the toxin was determined with HPLC-FD. The parameters on LC-MS instrument (3200 Q TRAP LC/MS/MS system, AB-SCIEX, Concord, Canada) were optimized with the authentic GTX5, prior to the analysis. The flow injection analysis was performed in mass range of *m/z* 200–500 and positive ion mode at 500 °C. The eluents were flowed at 0.2 mL/min with 65% B, where eluent A was distilled water and eluent B was acetonitrile, both containing 20 mM formic acid. The MS/MS analysis was carried out in mass range of *m/z* 50–450 at declustering potential of 11 V and collision energy of 21 V. The fragmentation pattern between the product and the authentic was compared and identified.

### 3.5. Preparation of GTX2/GTX3 from C1/C2 by Acidic Hydrolysis

Preparation of GTX2/GTX3 was carried out according to a previous method [12]. C1/C2 (1.4 μmol) were evaporated to dryness and dissolved in 1 mL of 0.13 M HCl aq. The solution was heated at 100 °C, stirring for 15 min and then cooled on ice. The products, GTX2/GTX3, were recovered using a ZIC-HILIC cartridge as described above. The reaction quantitatively proceeded. The structure was confirmed as described above.

## 4. Conclusions

In this study, major N1-H toxins were prepared from C1/C2 as starting material. In particular, this is the first report of the detailed conditions for preparation of GTX5 from C1/C2 analogues. The chemical conversion allows us to prepare the toxins systematically. The toxin yield, of over 220 μmol of C1/C2 extracted from 200 L of culture of the cyanobacteria, was estimated to be capable of preparing at least 10 μmol of each toxin, based on our reaction yields (Table 2). We previously reported a method using NMR to quantify concentrations of PST standards [23]. Based on the preparation technique of PSTs and the quantitative NMR, large amount of the N1-H toxin standards could be prepared and supplied constantly for shellfish safety monitoring programs using chemical analyses. The N1-OH toxins such as GTX1/GTX4 are also essential for the chemical analyses. The preparation technique of these toxins is currently being investigated, and will be reported elsewhere.

## Figures and Tables

**Table 1.** Biomass and toxin production of C1/C2 at 28 days of *A. circinalis* under modified condition of Fitzgerald medium.

**Entry**	**Parameters**		**Biomass**	**Toxin concentration of C1/C2 (μmol/L)**		
				**Total**	**Intracellular (%)**	**Extracellular (%)**
	Control		2300	1.39	80	20
**1**	NaNO_3_	0 mM	400	0.82	75	25
**2**		2.9 mM	1200	1.26	86	14
**3**		11.6 mM	1600	1.03	84	16
**4**	K_2_HPO_4_	0.14 mM	1600	1.43	86	14
**5**		0.54 mM	2000	1.49	84	16
**6**	Inoculation	0.5 L	2000	1.36	77	23
**7**		2 L	2500	1.05	84	16
**8**	Pyridoxal phosphate	10 μM	2500	1.36	58	42
**9**	Glycerophosphate Na	10 μM	3100	1.89	55	45
**10**		100 μM	2300	1.33	59	41
**11**	Bicine	0.6 mM	2700	1.78	3	97

	Average *			1.35	75	25

* Average toxin concentration was calculated using the data from entry 1 to 10.

**Table 2.** Amounts of toxins prepared from large-scale culture (200 L) of cyanobacteria *A. circinalis.* The amounts of C1/C2 as substrates are calculated as 110 μmol, because the toxins are isolated at 50% yield from the extract of the cyanobacteria.

**Reaction steps ^§^**	**Substrates**	**Amounts (μmol)**	**Products**	**Amounts (μmol)**	**Yields ^¶^ (%)**	**Remaining substrates**	**Residual Amounts (μmol)**	**Residual ratio ^†^ (%)**
**A**	C1/2	70	dcGTX2/3	35	50	C1/2	21	30
B *	dcGTX2/3	15	dcSTX	14	90			
**C**	C1/2	25	GTX5	12	48	C1/2	5	18
**D**	C1/2	15	GTX2/3	14	90			

§ The steps correspond to Figure 3;

* dcGTX2/3 were used as a part of the products in step A;

¶ (Amount of Product)/(Amount of Substrate) × 100;

† (Residual Amounts)/(Amount of Substrate) × 100.
